# CuS@BSA-NB2 Nanoparticles for HER2-Targeted Photothermal Therapy

**DOI:** 10.3389/fphar.2021.779591

**Published:** 2022-01-21

**Authors:** Ming Ying, Qin Li, Jingbo Wu, Yihang Jiang, Zhourui Xu, Mingze Ma, Gaixia Xu

**Affiliations:** ^1^ Guangdong Provincial Key Laboratory of Genome Stability and Disease Prevention, Shenzhen Key Laboratory of Marine Bioresources and Ecology/Shenzhen Key Laboratory of Microbial Genetic Engineering, College of Life Sciences and Oceanography, Shenzhen University, Shenzhen, China; ^2^ Guangdong Key Laboratory for Biomedical Measurements and Ultrasound Imaging, School of Biomedical Engineering, Health Science Center, Shenzhen University, Shenzhen, China

**Keywords:** CuS@BSA-NB2 nanoparticles, photothermal therapy, HER2, NB2, MDA-MB-231/HER2

## Abstract

Breast cancer is characterized by the uncontrolled proliferation of breast epithelial cells under the action of a variety of carcinogens. Although HER2-inhibitors were currently applied for HER2-positive breast cancer patients, they didn’t work for patients with resistance to HER2-targeted anti-cancer drugs. In this work, we prepared novel CuS@BSA-NB2 nanoparticles (NPs) for breast cancer photothermal therapy (PTT). The NPs had good biocompatibility due to the Bovine Serum Albumin (BSA) encapsulating and excellent targeting to HER2 because of nanobody 2 (NB2). Under 808 nm laser irradiation, CuS@BSA-NB2 NPs had high photothermal conversion efficiency and photothermal stability. Meanwhile, we constructed a stable cell line of MDA-MB-231/HER2 with a high expression of HER2 protein. Immunofluorescence and ICP-MS assays showed that CuS@BSA-NB2 NPs can be specifically enriched and be ingested in MDA-MB-231/HER2 cells. Furthermore, CuS@BSA-NB2 NPs had shown a more significant photothermal treatment effect than CuS@BSA under certain treatment conditions for MDA-MB-231/HER2. In addition, the cytotoxicity assay demonstrated that CuS@BSA-NB2 NPs had a low toxicity for MDA-MB-231/HER2 cells. The above results suggested that CuS@BSA-NB2 NPs were great photothermal therapeutic agents to reduce the malignant proliferation of breast epithelial cells and have potential for breast cancer therapy.

## Introduction

The latest statistical data has shown that female breast cancer surpassed lung cancer for the first time, and became the most common cancer in the world (Sung et al., 2021). Human epidermal growth factor receptor-2 (HER2) is an important prognostic factor of breast cancer. About 20–30% of breast cancer patients with overexpression of HER2 generate highly invasive, short disease-free survival and poor prognosis (Baselga, 2006). Therefore, HER2 was frequently used as the target in breast cancer targeted therapy (Oh and Bang, 2020). The anti-HER2 targeted drugs have greatly improved the therapeutic effect of HER2-positive breast cancer patients (Loibl and Gianni, 2017). Currently, HER2-targeted therapy drugs mainly include three categories: monoclonal antibodies as Trastuzumab, Pertuzumab (Howie et al., 2019) and Inituzumab and small molecules as Lapatinib, Pyrrotinib (Untch et al., 2018) and antibody-drug conjugates (ADCs) as T-DM1 (Li et al., 2016; Borges et al., 2018). In clinics, Pertuzumab and Trastuzumab combined with Docetaxel are the main first-line standard treatment for Her2-positive breast cancer (Gianni et al., 2012; Shao et al., 2020). Although monoclonal antibodies can bring substantial benefits, these will develop resistance and also have strong toxic side effects, such as cardiotoxicity (Telli et al., 2007). Hence, neoadjuvant chemotherapy is an important means for the treatment of breast cancer (Loibl et al., 2021). In order to establish HER2-targeted therapeutic effect, a nanobody (NB), namely the variable domain of heavy chain of heavy-chain (VHH) antibody, is a special antibody from camelid animals, which was applied to research owing to its advantages of higher stability, higher affinity, easier expression, and stronger tissue penetration compared with conventional antibodies (Teillaud, 2012; Muyldermans, 2013). Among these, a kind of HER2 nanobody (NB2) can specially recognize HER2 (Revets et al., 2008). In addition, the nanobody only contains a heavy chain variable domain, and the light chain is naturally missed, so there is only a single antigen-binding domain. Therefore, the nanobody can recognize the sites that is difficult for a conventional antibody to identify and access for the inclination of binding the concave epitopes as enzyme catalytic sites (De Genst et al., 2006). In summary, the nanobody has a huge application prospect in the treatment of tumors (Sun et al., 2021).

Biomedical nanoparticles (NPs) have advanced cancer therapy due to their high drug loading, low side effects, precise treatment, and excellent therapeutic effects (Yang et al., 2015; Meng et al., 2016). Among these, photothermal therapeutic nanoparticles have been continuously applied and developed owing to their small size, high thermal conductivity, large surface area, and so on. CuS NP (Copper Sulfide Nanoparticle) was a kind of photothermal therapeutic nanoparticle with the properties of plasmon resonance properties and possessed a high photothermal conversion efficiency in a short time especially under the irradiation of an external near-infrared laser (Wu et al., 2008). In addition, CuS NP was widely used in the detection of biomolecules, chemical substances, and pathogens *in vitro*, and cancer treatment and drug delivery *in vivo*, which had a broad potential for biomedical and clinical applications (Goel et al., 2014). Tumors treated by CuS NP can locally warm up and ablate for the qualities of photothermal and photodynamic (Dong et al., 2013). Due to the diversification of treatment methods and material structure properties, the combination of CuS NP with other special drugs as clinical radiotherapy and chemotherapy drugs or molecules is generally designed and synthesized for cancer therapy, which reveals better effects than CuS NP alone (Marianne et al., 2017; Dai et al., 2018; Goel et al., 2018; Jauffred et al., 2019; Xiao et al., 2020). Therefore, the design of CuS nanoparticles with NB2 is expected to achieve photothermal therapy of breast cancer according to the function of targeting HER2.

**FIGURE 1:**
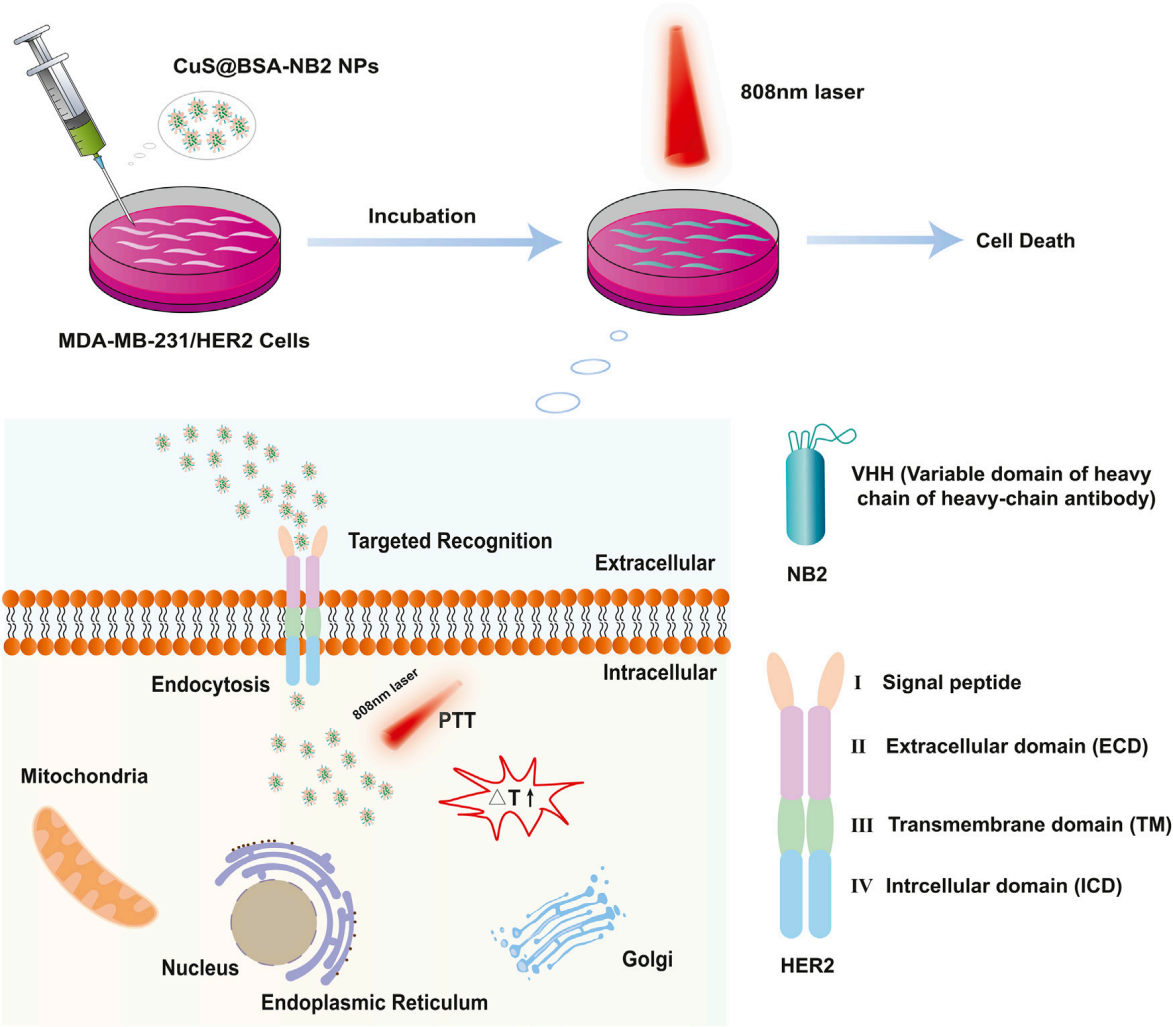
Schematic diagram of CuS@BSA-NB2 NPs targeting HER2 for photothermal therapy in MDA-MB-231/HER2 cells.

In this work, we prepared a novel biocompatible nanoparticle CuS@BSA-NB2 which had two functions: targeting cancer cells with high expression of HER2 protein, and killing breast cancer by photothermal effect (Figure 1). Firstly, we purified and verified NB2, then synthesized CuS@BSA-NB2 NPs. Then Transmission Electron Microscopy (TEM), Dynamic Light Scattering (DLS), Ultraviolet-visible Spectrophotometry, Fourier transform infrared spectroscopy, and infrared thermography were used to characterize nanoparticles. In addition, we constructed a stable strain with a high expression of HER2 in MDA-MB-231 cells. Western blot and immunofluorescence assays were used to analyze the successful construction of cell lines. Then, immunofluorescence and Inductively Coupled Plasma Mass Spectrometry (ICP-MS) experiments were used to verify that CuS@BSA-NB2 NPs can specifically target MDA-MB2-231/HER2 cells and the cells can specifically ingest nanoparticles. Finally, we verified cytotoxicity and phototoxicity of CuS@BSA-NB2 NPs, and the AM and PI assay was used to verify the specific photothermal treatment of MDA-MB-231/HER2 cells with CuS@BSA-NB2 NPs. These results indicated CuS@BSA-NB2 NPs as photothermal therapeutic nanoparticles that have a great potential for breast cancer treatment.

## Methods and Materials

### Cell Culture

MDA-MB-231 cells were obtained from American Type Culture Collection (ATCC, United States). The cells were cultured in Dulbecco’s modified Eagle’s medium (DMEM, HyClone™, United States) containing 10% fetal bovine serum (FBS, HyClone™, United States) and 1% 100 U/mL penicillin/streptomycin (Solarbio, Beijing, China), and maintained at 37 °C in a humidified atmosphere incubator (Yamato, Japan) with 5% CO_2_.

### Construction of Plasmid

The target gene was constructed into the lentiviral empty vector by polymerase Chain Reaction (PCR). After the exogenous Deoxyribonucleic Acid (DNA) was extracted by PCR, the empty vector and the exogenous DNA fragments were cut with restriction endonuclease respectively for 2 h at 37°C, and they were connected by DNA ligase overnight at 16°C, and then transferred into DH5α. Finally, the recombinant clones were obtained through screening and identification. The HER2 (Gene ID: NM_004448.4) was constructed into pLV3, and the HER2 extracellular domain (HER2 ECD) from 23rd amino acid to 652nd amino acid of HER2 was constructed into pET-28a. The primers of HER2 and HER2 ECD are shown in Supplementary Table S1.

### Construction of Stable Cell Line

The pLV3-HER2 and pLV3 were constructed into the MDA-MB-231 cells respectively. Plasmids (6 μg) (psPAX2:pMD2. G:plv3-HER2/plv3 = 3:1:4) were added to 500 μL of serum-free DMEM and stood for 5 min. Then transfection reagent (18 μg) was added into it and stood for 20 min. The mixture and medium (1.5 ml) were added into a 6 cm dish with 293T cells of 80% density, and cultured for 8 h. Then fresh medium (5 ml) was added into the dish for 30 h, and collected to be the virus. Subsequently, virus (1 ml), fresh medium (1 ml), and polybrene (8 μg/ml) were added into a 35 mm dish with MDA-MB-231 cells of 40% density twice per 24 h for infecting the cells. Finally, the medium with puromycin (10 μg/ml) was added into the dish to kill the uninfected cells until the cells no longer died, which were the stable cell lines. Finally, the stable cell lines were taken to be centrifuged (5,000 rpm, 5 min, 4°C), then resuspended by PBS (100 μl) and added 40 μl 6 × western blot loading buffer to be heated for 20 min at 100°C to become the cell lysates for analysis.

### Synthesis of CuS@BSA NPs

CuS@BSA NPs were prepared according to the wet chemical method (Wan et al., 2019). Firstly, CuCl_2_·2H_2_O (MACKLIN, China) (0.75 M, 250 μl) was added to the aqueous BSA (Yancheng Saibao Biotechnology Co., Ltd., China) solution (10 mg/ml, 10 ml) dropwise in the round-bottom flask (25 ml) under magnetic stirring at room temperature for 10 s, and the solution turned blue. Then 109 μl Ammonium sulfide solution (H_8_N_2_S) (MACKLIN, China) was added to the mixture for 10 s, and the solution turned brown. The mixture was placed into an oil bath and stirred for 30 min, and placed at room temperature naturally after the reaction was finished. The mixture was then resuspended by PBS (6 ml) after being ultra-filtered (MWCO = 100 kDa) for 20 min at 4,000 rpm, and filtered with 0.22 μm syringe after being dialyzed (MWCO = 1,000 kDa) in PBS for 24 h at 4°C. Finally, CuS@BSA NPs were stored at 4°C.

### Induced Expression and Purification of NB2

The *Escherichia coli* BL21-DE3 with recombinant plasmid carrying the NB2 gene came from our own laboratory. Glycerol bacteria (10 μl) were added into LB medium (20 ml) with 50 μg/ml ampicillin to be cultured at 37°C overnight in a shaker. The seed medium was inoculated to 250 ml ampicillin-resistant LB medium, and the ratio of inoculation was 1%. When the OD_600_ value reached 0.6–0.8, 1 ml medium was taken as the pre-induced sample. Then 1 ml medium was taken as the induced sample after the medium was induced overnight at 19°C by isopropyl-β-d-thiogalactoside (IPTG, 0.5 M). The medium was then centrifuged (4,000 rpm, 15 min, 4°C), and the supernatant was discarded, and the cells were stored at −80°C overnight. PBS (30 ml) with 1 mM Phenylmethanesulfonyl fluoride (PMSF) (Sigma-Aldrich, United States) was added to resuspend the bacteria on the second day and sonicated at the following settings: turning on for 2 s and off for 3 s, 30% power, 30 min Then the bacteria were centrifuged (5,000 rpm, 10 min, 4°C), and the supernatant was collected. Meanwhile, some of the deposit and 100 μl supernatant were taken as the samples to be tested. The supernatant was added to pass through the column with 1 ml Glutathione Resin (L00206, GenScript, China) after being washed by 10x volume of PBS in advance, and 100 μL solution was taken as the outflowed sample. Then 20x volume of PBS was used to remove other proteins, and 100 μl solution was taken as the washed sample at the same time. l-Glutathione (Reduced) (G8180, Solarbio, China) (10 mM, pH = 8.0) was used to elute NB2, and 100 μl solution was taken as the eluted sample. Finally the NB2 was dialyzed (MWCO = 14 kDa) in PBS for 24 h, then centrifuged (1,000 rpm, 10 min, 4°C), and stored at −80°C. Among these, the samples with bacteria were washed three times with PBS for 5 min every time at 5,000 rpm, 4°C, and added 100 μL PBS to be resuspended. Then all samples were added 40 μl 6 × western blot loading buffer respectively to be prepared the protein samples for analysis after being heated for 20 min at 100°C.

### Transfection of Plasmid

First, pET-28a-HER2 (1 μl) plasmids were added into BL21-DE3 (50 μl) for 30 min on ice, then incubated in a water bath of 42°C for 2 min, and stood on ice for 3 min. LB medium (750 μl) was added, then it was incubated for 1 h at 37°C in the shaker. The bacteria were centrifuged (5,000 rpm, 1 min, 4°C), and resuspended by the medium after 500 μl medium was removed. Then the solution was added to the ampicillin-resistant LB solid medium containing glass beads. The plate was incubated overnight at 37°C.

### Induced Expression of HER2 ECD

After pET-28a-HER2 ECD plasmids were transfected into BL21-DE3 as above, and three colonies were added to 4 ml ampicillin-resistant LB liquid medium respectively to be incubated overnight at 37°C in a shaker. The seed medium was inoculated to 25 ml ampicillin-resistant LB medium, and the ratio of incubation was 1%. When the OD_600_ value reached 0.6–0.8, 1 ml medium was taken as the pre-induced sample. Then 1 ml medium was taken as the induced sample after the medium was induced overnight at 18°C by 0.5 M IPTG. The medium was then centrifuged for 15 min at 4°C, 4,000 rpm, and the supernatant was discarded. The bacterias were stored at −80°C overnight. 5 ml PBS with 1 mM PMSF was added to resuspend the bacteria on the second day, and sonicated at the following settings: turning on for 2 s and off for 3 s, 30% power, 30 min. Then the bacteria were centrifuged (5,000 rpm, 10 min, 4°C), and the supernatant was collected. Meanwhile, some of the deposit and 100 μl supernatant were taken as the samples to be tested. All samples were treated as above to be prepared the protein samples for analysis.

### Pull Down

The interaction of HER2 ECD protein and NB2 was detected by pull-down. Ni NTA Beads 6FF (SA005010, Smart-Lifesciences, China) of 30 μl were added to two EP tubes and washed three times with 1 ml binding buffer for 5 min at 4°C, 5,000 rpm. One was treated with 1 ml pET-28a-HER2 (ECD) supernatant, 1 mM PMSF, and 20 μl NB2 protein, and the other was treated the same, but with NB2 protein overnight at 4°C. The beads were centrifuged (5,000 rpm, 5 min, 4 °C), and washed three times with 1 ml wash buffer each by centrifuged (5,000 rpm, 5 min, 4°C). The beads were treated as above to become the protein samples for analysis.

### SDS-PAGE

The protein samples were analyzed by 10% SDS-PAGE. Then the gel was dyed by coomassie brilliant blue R250 solution at room temperature for 30 min at 80 rpm, and then decolored by decolorizing solution (10% acetic acid, 30% methanol) at room temperature for 30 min at 80 rpm. The experiments were tested by the same method as above as induced expression and purification of NB2, and the induction and expression of HER2 (ECD) and pull down of NB2 and HER2 (ECD).

### Western Blot

The related proteins were detected and analyzed by western blot. Firstly, the prepared protein samples and markers were loaded into the polyacrylamide gel for electrophoresis. When the electrophoresis was completed, the protein was transferred to the polyvinylidene fluoride (PVDF) (IPVH00010, Sigma-Aldrich, China) wetted by methanol. The blot was incubated with 5% skimmed milk at room temperature, then incubated with diluted primary antibody overnight at 4°C. The next day, the blot was washed three times with TBST for 4 min each next day and incubated with the corresponding HRP-conjugated second antibody for 40 min at room temperature. The blot was then washed as before. Finally, the blot was developed after being added UltraSignal ultra-sensitive electrochemiluminescence substrate (4AW011-500, 4 A Biotech, China).

The following experiments were based on the above method. For the experiment of the purification of NB2, GST antibody (1:1,000) was the primary antibody. For the induction and expression of HER2 (ECD) protein, His tag antibody (66005-1-Ig, Proteintech, United States, 1:1,000) was the primary antibody. For the experiment of NB2 recognizing HER2 (ECD) protein, GST antibody (1:1,000) was the primary antibody, and NB2 (1:1,000) was the secondary antibody and the secondary antibody of GST antibody was the third antibody. For the pull-down experiment, His antibody (1:1,000) and GST antibody (1:1,000) as primary antibodies were added to incubate the membrane solely. For the detection of stable cell lines, FLAG antibody (F1804, Sigma, United States, 1:5,000), HER2 antibody (2165S, Cell Signaling Technology, United States, 1:1,000), and β-actin antibody (#P30002, Ab-mart, China, 1:5,000) as the primary antibodies were added to incubate the membrane respectively.

### Synthesis of CuS@BSA-NB2 NPs

CuS@BSA NPs (1.2 mg/ml, 1 ml) were resuspended by MES (M8010, Solarbio, China) buffer (0.1 M, pH = 6.0, 1 ml) after being ultra-filtered (MWCO = 100 kDa) (4,400 rpm, 20 min). 1-ethyl-3-(3-dimethylaminopropyl) carbodiimide hydrochloride (EDC) (E106172-25g, Aladdin, China) of 0.4 and 0.7 mg N-Hydroxysuccinimide (NHS) (H109330-25g, Aladdin, China) were then added into the CuS@BSA NPs solution for 30 min. The CuS@BSA NPs were then ultra-filtered (MWCO = 100 kDa) for 20 min at 4,400 rpm to remove redundant EDC and NHS and resuspended by PBS (1 ml). 100 μl of NB2 (4 mg/ml) was added to the solution of CuS@BSA NPs slowly to response for 2 h. Finally, CuS@BSA-NB2 NPs were dialyzed (MWCO = 100 kDa) for 24 h at 4°C to remove excessive NB2 and stored at 4°C ultimately.

### Characterization of Nanoparticles

The TEM images of CuS@BSA-NB2 NPs and CuS@BSA NPs were obtained by a tungsten filament transmission electron microscope HITACH HT7700 instrument at an acceleration voltage of 80 kV. The size distribution images were obtained by a dynamic light scattering particle size analyzer (Zetasizer Nano ZS, Malvern, United Kingdom). The ultraviolet-visible absorption spectrum data was measured by an ultraviolet-visible spectrophotometer (Lambda 750, PE, United States). The infrared absorption data was measured by Fourier infrared spectrometer (Nicolet 6,700). The temperature-time curve data and the *in vitro* infrared imaging images were measured by an 808 nm near-infrared laser and infrared measured with an imager (FL-IR A300-Series, Sweden).

### Photothermal Property of the CuS@BSA-NB2 NPs

In order to test the efficiency of photothermal conversion of CuS@BSA-NB2 NPs, which were diluted by PBS to different concentrations (0 μg/ml, 12.5 μg/ml, 25 μg/ml, 50 μg/ml, 100 μg/ml, 200 μg/ml), and irradiated for 4 min respectively under 808 nm (1 W/cm^2^). In order to detect the photothermal stability of nanoparticles, 200 μg/ml CuS@BSA NPs and CuS@BSA-NB2 NPs were measured under 808 nm (1 W/cm^2^) respectively. The laser was turned on for 2 min, then turned off to be cooled to room temperature for four cycles. In addition, 200 μg/ml CuS@BSA NPs and CuS@BSA-NB2 NPs were irradiated for 10 min under the same condition to observe the change of temperature with images at different times.

### Immunofluorescence Assay

The stable cell lines were detected by immunofluorescence. MDA-MB-231/HER2 cells and MDA-MB-231-EV cells were seeded into a 35 mm dish with respectively for being incubated overnight, and the cell density was about 60–70%. On the second day, the medium was discarded, and each dish was washed with PBS three times. Paraformaldehyde (4%) of 1 ml was then added to the per dish for 15 min. Then 1% TritonX-100 (500 μl) was added to the per dish to incubate for 10 min. The 100 μl primary antibody sample containing FLAG antibodies (1:100) and HER2 antibodies (1:100) was then added to incubate the cells for 1 h. Then 100 μl secondary antibody sample containing Alexa Fluor 488-conjugated Affinipure Goat Anti-Mouse IgG (H + L) (SA00006-1, proteintech, United States, 1:250) and Rhodamine (TRITC)-conjugated Goat Anti-Rabbit IgG (H + L) (SA00007-2, proteintech, United States, 1:250) was added to incubate for 30 min in dark. Then 1 ml DAPI (1:5,000) was added to incubate for 10 min in dark. Among these, each dish should be washed three times by PBS after the above reagents were added. Finally, the cells were observed and photographed under a fluorescence microscope.

The CuS@BSA-NB2 NPs targeting MDA-MB-231/HER2 cells were detected by immunofluorescence. The two kinds of cells were seeded into a 12-well plate with a cell density of about 60–70%. Medium (1 ml) with CuS@BSA-NB2 NPs (1.2 mg/ml, 10 μl) were then added to each well and incubated for 2 h. Next, the operation of immunofluorescence was performed similarly as above. In this work, the NB2 in CuS@BSA-NB2 NPs were the primary antibodies, and the MonoRab™ Rabbit Anti-Camelid VHH Cocktail (iFluor 647) (A02019-200, GenScript, China, 1:1,000) were the secondary antibodies. Finally, the cells were observed and photographed under Ultra-high resolution confocal microscopy (LSM880, CARL ZEISS, Germany).

### Cellular Uptake of Nanoparticles

In order to verify whether there was a difference in the content of CuS@BSA-NB2 NPs and CuS@BSA NPs uptake in MDA-MB-231/HER2 cells, Inductively Coupled Plasma Mass Spectrometry (ICP-MS) (Agilent 7,700, United States) was used to detect the Cu amount of cellular uptake. MDA-MB-231/HER2 cells were added to three 6-well plates to be cultured. After the cell density reached 80%, the original medium was removed and replaced with 50 μg/ml CuS@BSA NP and CuS@BSA-NB2 NP media respectively. Then the cells were incubated for 0, 12, and 24 h respectively. In each time period, each well was washed with PBS three times before the medium of each well was removed and added 300 μl trypsin (0.25%) for 3 min to digest the cells. After the digestion was complete, 1 ml medium was added to stop the digestion and the cells were collected by centrifuge (5,000 rpm, 5 min) respectively. Then the cells were resuspended and washed by PBS after the medium was removed, and centrifuged (5,000 rpm, 5 min). Then 500 μl Nitric acid (65%) (Huachengda Chemical Co., Ltd., Zhuhai, China) was added respectively to resuspend the cells to completely digest the cells and decompose the nanoparticles into ions at 37 °C overnight. Finally, the samples were sent to Guangzhou Chemical Union Quality Testing Technology Co., LTD. to detect the concentration of Cu in the cell digestion solution.

### MTT Assay for Cytotoxicity and Phototoxicity

The cytotoxicity of CuS@BSA-NB2 NPs was measured by MTT assay. MDA-MB-231/HER2 cells were seeded into a 96-well plate with 100 μl medium containing about 5,000 cells per well for 24 h. CuS@BSA-NB2 NPs were diluted to varied concentrations by medium (0 μg/ml, 12.5 μg/ml, 25 μg/ml, 50 μg/ml, 100 μg/ml and 200 μg/ml). A 100 μl volume of medium with various concentrations of nanoparticles was added to each well for 24 h. The medium was then removed, and serum-free DMEM (100 μl) containing 3-(4,5-dimethyl-thiazol-2-yl)-2,5-diphenyltetrazolium bromide (MTT) (10 μl) was added to each well for 4 h after being washed three times with PBS. Finally, DMSO (150 μl) was added to each well after the supernatant was removed to measure the absorbance at 570 nm by the multifunction microplate reader (bioTek, United States).

CuS@BSA-NB2 NPs at 50 μg/ml were selected to detect phototoxicity. The MDA-MB-231/HER2 cells were seeded in a 96-well plate with a cell density of 80% and divided into two equal groups. A 100 μl volume of medium with PBS, CuS@BSA NPs, or CuS@BSA-NB2 NPs were added to incubate per well for 24 h. After that, one group was irradiated with 808 nm (80 s, 2 W/cm^2^), and the other group was not irradiated as a control, then cultured for 2 h. Finally, the absorbance was detected by MTT assay as above.

### AM/PI Assay for Photothermal Therapy

CuS@BSA-NB2 NPs at 50 μg/ml were used to test photothermal therapy. The MDA-MB-231/HER2 cells were seeded in a 24-well plate with a cell density of 80% and divided into three equal groups to be irradiated or unirradiated. Each group was treated by PBS, CuS@BSA-NB2 NPs, or CuS@BSA NPs for 24 h. After that, two groups were irradiated with 808 nm (160 s, 1 W/cm^2^) and 808 nm (80 s, 2 W/cm^2^), and another group was not irradiated as a control, and cultured for 2 h. The medium was removed, then fresh medium with 2 μM Calcein Acetoxymethyl Ester (AM) (C2012-0.1ml, Beyotime, China) was added to each well for 30 min. 50 μg/ml Propidium Iodid (PI) (81,845-100 MG, Sigma-Aldrich, United States) was added for 10 min after the AM was removed. Each well was then added after being removed the PI and washed with PBS. Finally, an inverted fluorescence microscope was used to observe the alive and dead cells.

### Statistical Analysis

The data are presented as mean ± SD. Student’s *t*-test was used to measure statistical differences between groups and the value of *p* < 0.05 was considered statistically significant.

## Results

### Synthesis of CuS@BSA NPs and Identification of NB2

**FIGURE 2:**
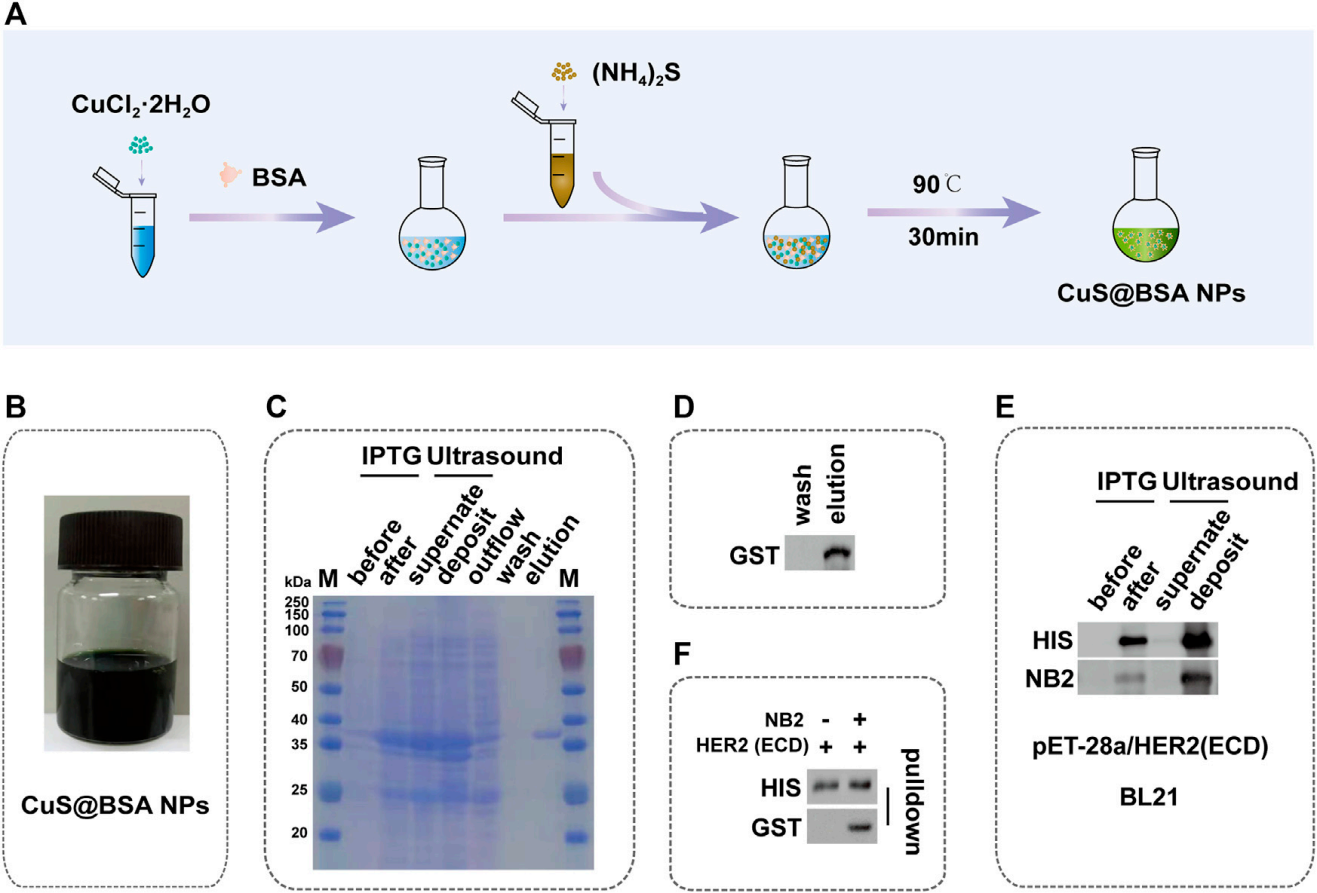
Synthesis of CuS@BSA NPs and Identification of NB2. **(A)** Schematic diagram of the synthesis process of CuS@BSA NPs. **(B)** The synthesized solution of CuS@BSA NPs. **(C)** SDS-PAGE image of the expression and purification of NB2. The molecular weight of NB2 was 38 kDa showed in the elution lane. M: marker. **(D)** Western blot of NB2 expression and purification. **(E)** Western blot of induction and expression of HER2 ECD protein in BL21-DE3 and the NB2 recognizing HER2 ECD protein. **(F)** Western blot of NB2 and supernatant of being induced and broken of HER2 ECD protein co-incubation.

CuS@BSA NPs were prepared by the wet chemical method (Figure 2A), and the color of the final solution was dark green (Figure 2B). The molecular quantity of the purified NB2 was about 38 kDa by SDS-PAGE electrophoresis (Figure 2C). At the same time, the NB2 was purified successfully through the eluent through western blot analysis (Figure 2D). In order to verify whether NB2 can recognize the HER2 extracellular domain (HER2 ECD) protein, firstly, the HER2 ECD protein was analyzed to express successfully in BL21-DE3 (Figure 2E, Supplementary Figure S1). Afterward, NB2 was used as the primary antibody to identify HER2 ECD protein, and then the GST tag antibody was used as the secondary antibody to recognize NB2. Finally, we found that NB2 can exactly recognize the HER2 ECD protein (Figure 2E). In addition, NB2 could also interact with the HER2 ECD protein by pull-down (Figure 2F, Supplementary Figure S2).

### Characterization of Nanoparticles

**FIGURE 3:**
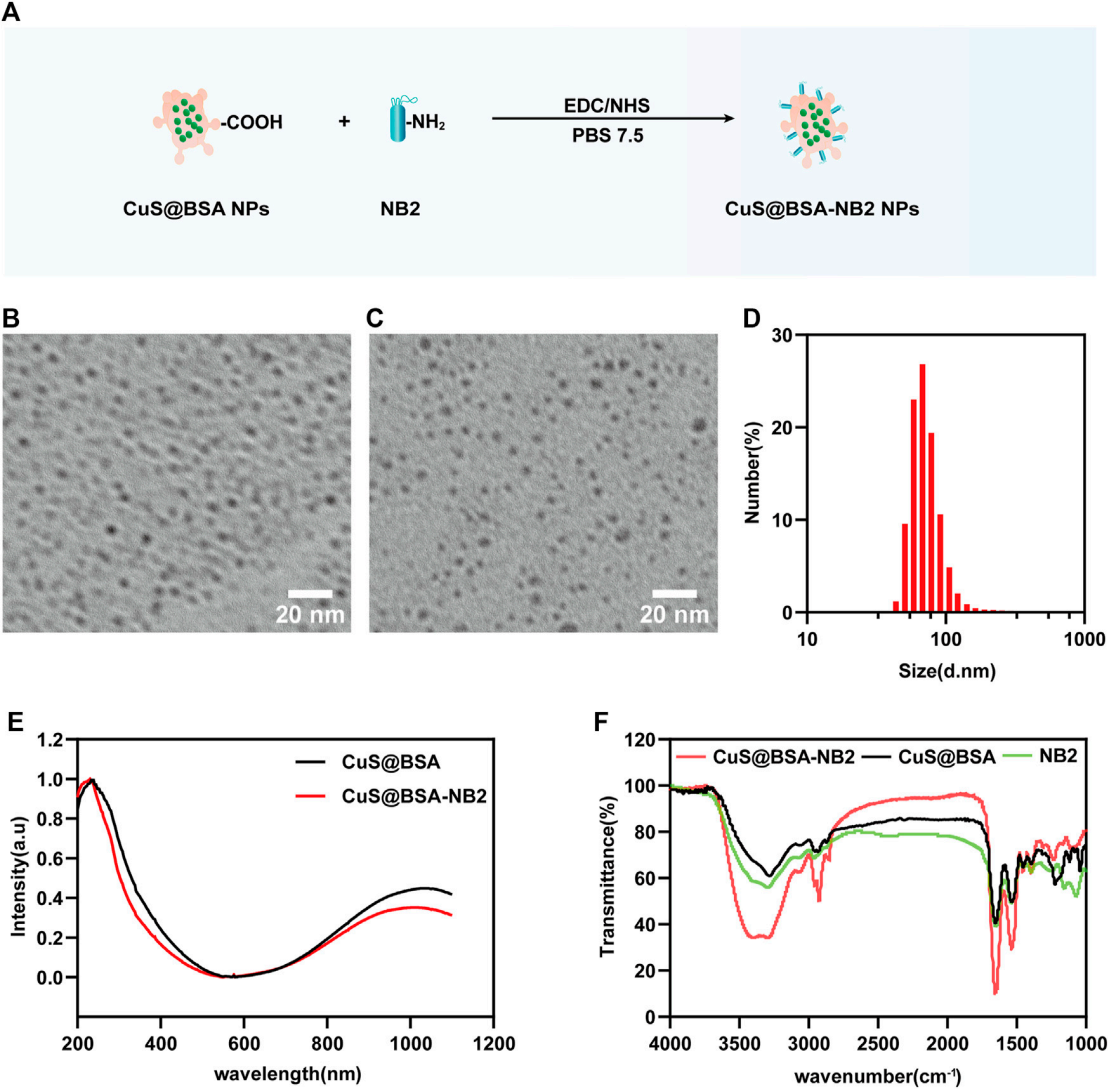
Characterization of nanoparticles. **(A)** Schematic diagram of the process of biosynthesis of CuS@BSA-NB2 NPs. **(B) **TEM image of CuS@BSA NPs, and the scale bar was 20 nm. **(C)** TEM image of CuS@BSA-NB2 NPs, and the scale bar was 20 nm. **(D)** DLS analysis of CuS@BSA-NB2 NPs. **(E)** UV-vis absorption curves of CuS@BSA NPs and CuS@BSA-NB2 NPs. **(F)** Fourier infrared spectrum absorption curves of CuS@BSA, CuS@BSA-NB2 and NB2.

We conjugated CuS@BSA NPs and NB2 with EDC and NHS to form peptides to synthesize CuS@BSA-NB2 NPs (Figure 3A). In order to better understand the shape and size of CuS@BSA-NB2 NPs and CuS@BSA NPs, we have observed the TEM images of CuS@BSA NPs (Figure 3B) and CuS@BSA-NB2 NPs (Figure 3C) with a uniform distribution of about 6–8 nm. Furthermore, the average particle size of CuS@BSA NPs was about 18 nm (Supplementary Figure S3), and CuS@BSA-NB2 NPs was about 67 nm by dynamic light scattering (DLS) analysis (Figure 3D). Therefore, CuS@BSA-NB2 NPs were larger than CuS@BSA NPs due to the coupling of NB2. In addition, both CuS@BSA NPs and CuS@BSA-NB2 NPs had an absorption intensity of 1,052 nm, in the wavelength range of 200–1,100 nm (Figure 3E), which indicated that the nanoparticles can perform photothermal effects under near-infrared laser irradiation. Moreover, CuS@BSA-NB2 NPs had a stronger peak than CuS@BSA NPs because NB2 is in the wavenumber range of 3,000–3,500 cm^−1^, and CuS@BSA-NB2 NPs had formed more amide bonds under the coupling agent of EDC and NHS (Figure 3F). Therefore, the NB2 was conjugated to CuS@BSA successfully through FT-IR analysis (Yan et al., 2018).

### Photothermal Effect and Stability of CuS@BSA-NB2 NPs

**FIGURE 4:**
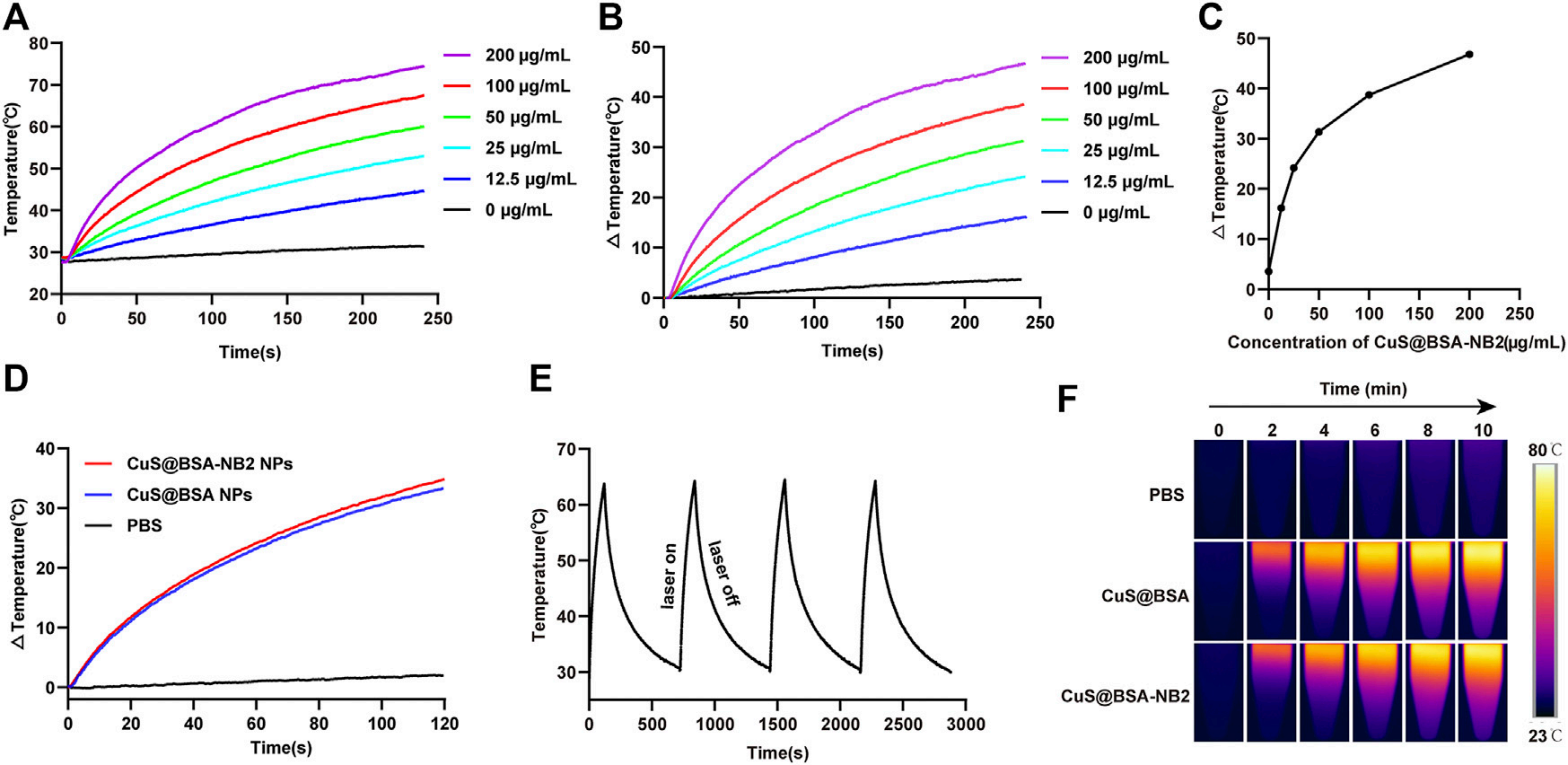
Photothermal effect and stability of CuS@BSA-NB2 nanoparticles. **(A)** The temperature curves of CuS@BSA-NB2 NPs with different concentrations irradiated with 808 nm (1 W/cm^2^, 4 min). **(B)** The temperature difference curves of CuS@BSA-NB2 NPs solutions with different concentrations over time irradiated with 808 nm (1 W/cm^2^, 4 min). **(C)** The temperature difference of CuS@BSA-NB2 NPs with different concentrations under 808 nm (1 W/cm^2^, 4 min). **(D)** The temperature difference curves of CuS@BSA NPs (200 μg/ml) and CuS@BSA-NB2 NPs (200 μg/ml) under 808 nm (1 W/cm^2^, 2 min). **(E)** The temperature curve of CuS@BSA-NB2 NPs (200 μg/ml) under 808 nm (1 W/cm^2^) rose and cooled for four cycles. **(F)** Infrared images of PBS, CuS@BSA NPs (200 μg/ml) and CuS@BSA-NB2 NPs (200 μg/ml) irradiated by 808 nm (1 W/cm^2^) at different times.

In order to verify that CuS@BSA-NB2 NPs had the proposed ability of light-to-heat conversion and stability under near-infrared laser radiation, we detected nanoparticles with an 808 nm laser and an infrared imager. CuS@BSA-NB2 NPs were diluted with PBS to different concentrations of 0 μg/ml, 12.5 μg/ml, 25 μg/ml, 50 μg/ml, 100 μg/ml, and 200 μg/ml, and irradiated for 4 min respectively under 808 nm (1 W/cm^2^) at room temperature. In this work, we found that the temperature of different concentrations of nanoparticles increased following the increment of concentrations of nanoparticles (Figure 4A). Furthermore, the temperature of 50 μg/ml CuS@BSA-NB2 NPs could reach 59°C at 808 nm (1 W/cm^2^, 4 min), which is over the 42°C minimum temperature for killing cells (Andrä and Nowak, 1998) (Figure 4A). The temperature difference curves of CuS@BSA-NB2 NPs solutions with different concentrations also increased over time under 808 nm (1 W/cm^2^, 4 min) (Figure 4B). Besides, the temperature difference increased with the different concentrations of nanoparticles (Figure 4C). Clearly, the greater the concentration of nanoparticles, the higher the temperature difference. Compared with the same concentration of CuS@BSA NPs, Cus@BSA-NB2 NPs also showed a similar warming trend under 808 nm (1 W/cm^2^, 2 min) (Figure 4D). In addition, in order to verify the photothermal stability of CuS@BSA-NB2 NPs, 200 μg/ml CuS@BSA-NB2 NPs were irradiated to heat up at 808 nm (1 W/cm^2^, 2 min) and non-irradiated to cool to room temperature. This process was repeated for four cycles (Figure 4E). The result showed that the photothermal conversion ability of CuS@BSA-NB2 NPs will hardly abate after continuous irradiation, which indicated that CuS@BSA-NB2 NPs had strong photothermal stability. Besides, CuS@BSA NPs had a similar situation (Supplementary Figure S4). Furthermore, the infrared images of CuS@BSA NPs and CuS@BSA-NB2 NPs showed a similar increase of temperature under the infrared imager under 808 nm (1 W/cm^2^) at the time of 0, 2, 4, 6, 8, and 10 min, but the PBS group showed a very small change of temperature single (Figure 4F). Therefore, CuS@BSA-NB2 NPs have high photothermal effect and stability after repeated irradiation.

### Specificity of CuS@BSA-NB2 NPs to HER2

**FIGURE 5:**
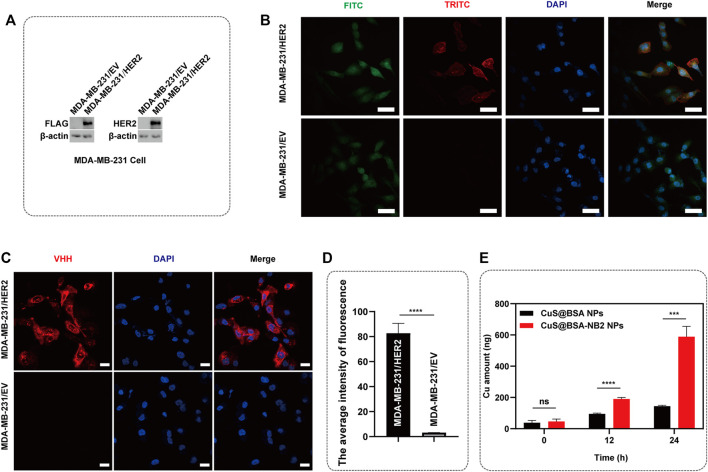
Specificity of CuS@BSA-NB2 NPs to HER2. **(A)** The western blot of the stable cell strains were constructed into MDA-MB-231 cells. **(B)** Immunofluorescence images of MDA-MB-231/HER2 cells and its control group. The scale bar was 50 μm. **(C)** Immunofluorescence images of MDA-MB-231/HER2 cells and its control group treated with CuS@BSA-NB2 NPs. The scale bar was 20 μm. **(D)** The average intensity of VHH fluorescence (*n* = 6, ****p < 0.001). **(E)** The cellular uptake Cu amount of MDA-MB-231/HER2 cells treated by 50 μg/ml CuS@BSA NPs and CuS@BSA-NB2 NPs respectively for 0, 12 and 24 h (*n* = 3, ****p < 0.0001, ***p < 0.001).

For certificating CuS@BSA-NB2 NPs can target HER2, we constructed an MDA-MB-231/HER2 cell line with high expression of HER2 protein in MDA-MB-231 cells. Besides, the lysate of the MDA-MB-231/HER2 cells and its control group showed that the MDA-MB-231/HER2 cells were successfully constructed by western blot (Figure 5A). In addition, MDA-MB-231/HER2 cells showed significant fluorescence intensity through immunofluorescence under fluorescent secondary antibodies TRITC and FITC respectively (Figure 5B). Next, MDA-MB-231/HER2 cells and control cells were treated with CuS@BSA-NB2 NPs and then subjected to immunofluorescence analysis. Under VHH antibody labeled iFluor-647, the MDA-MB-231/HER2 cells treated by CuS@BSA-NB2 NPs had significant fluorescence intensity, indicating that CuS@BSA-NB2 NPs can specifically target and recognize MDA-MB-231/HER2 cells better than the control group with lower HER2 expression (Figures 5C,D). Furthermore, we also detected the MDA-MB-231/HER2 cellular uptakes of CuS@BSA NPs and CuS@BSA-NB2 NPs respectively through ICP-MS assay to verify the specificity of CuS@BSA-NB2 NPs to HER2 high expression cells. The result showed that the Cu content of CuS@BSA-NB2 NPs uptake by MDA-MB-231/HER2 cells for 24 h was up to 588.68 ng, which was 4.08 times higher than CuS@BSA NPs (Figure 5E). Besides, the cellular uptake Cu amount was time-dependent. Therefore, CuS@BSA-NB2 NPs can specially recognize MDA-MB-231/HER2 cells and encourage the cells to take in more nanoparticles.

### Photothermal Therapy Effects of CuS@BSA-NB2 NPs on MDA-MB-231/HER2 Cells

**FIGURE 6:**
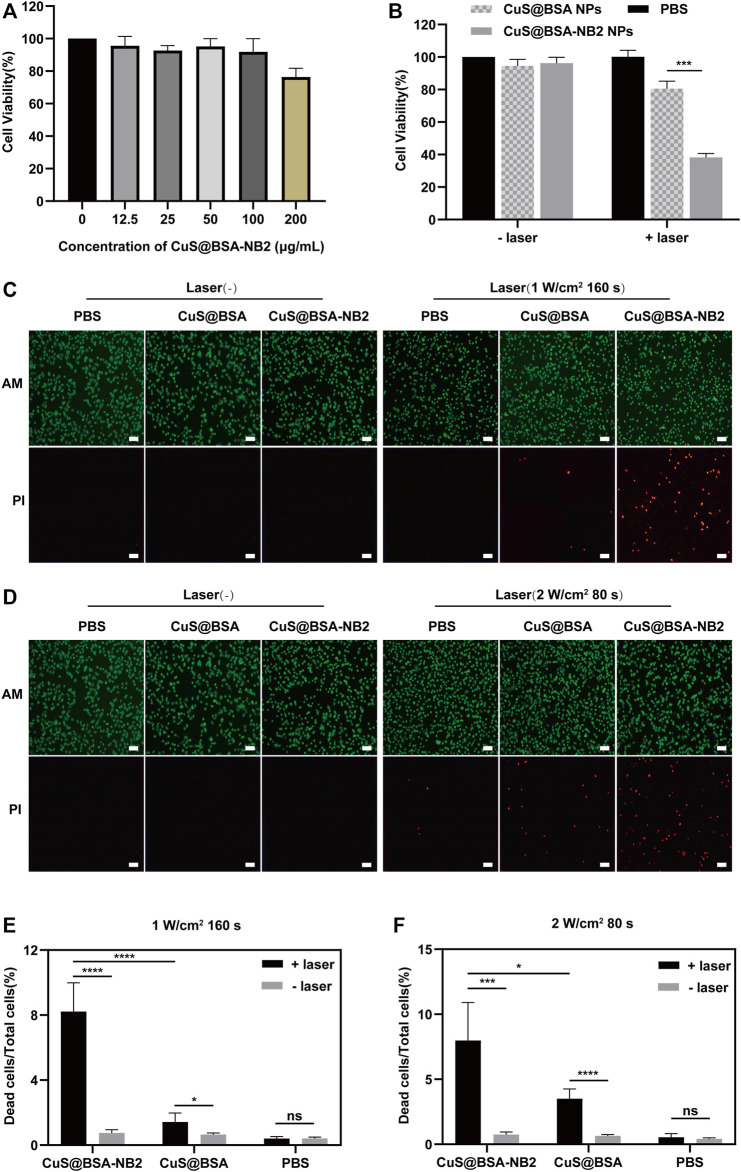
The photothermal effect of CuS@BSA-NB2 nanoparticles in the cell. **(A)** The cytotoxicity measured by MTT of CuS@BSA-NB2 nanoparticles at different concentrations incubated in MDA-MB-231/HER2 cells (*n* = 3). **(B)** MTT assay detected the photothermal effect of MDA-MB-231/HER2 cells treated with 50 μg/ml CuS@BSA-NB2 NPs and CuS@BSA NPs under 808 nm (2 W/cm^2^, 80 s) (*n* = 3, ***p < 0.01). **(C)** Living and dead cells stained by AM and PI images of MDA-MB-231/HER2 cells treated with 50 μg/ml CuS@BSA NPs and CuS@BSA-NB2 NPs and irradiated by 808 nm (1 W/cm^2^, 160 s). The scale bar was 100 μm. **(D)** Living and dead cells stained by AM and PI images of MDA-MB-231/HER2 cells treated by 50 μg/ml CuS@BSA NPs and CuS@BSA-NB2 NPs and irradiated under 808 nm (2 W/cm^2^, 80 s). The scale bar was 100 μm. **(E)** The data statistical analysis chart of the proportion of dead cells to total cells of Figure C (*n* = 5, ****p < 0.01). **(F)** The data statistical analysis chart of the proportion of dead cells to total cells of Figure D (*n* = 5, *p < 0.01).

In order to observe the cytotoxicity of CuS@BSA-NB2 NPs, MDA-MB-231/HER2 cells were incubated with different concentrations of CuS@BSA-NB2 NPs for 24 h, and the MTT assay revealed that CuS@BSA-NB2 NPs had almost no cytotoxicity (Figure 6A). Then, we tested whether CuS@BSA-NB2 NPs had a photothermal therapeutic effect in MDA-MB-231/HER2 cells. Firstly, 50 μg/ml of CuS@BSA NPs and CuS@BSA-NB2 NPs were used to treat MDA-MB-231/HER2 cells separately for 24 h. Under irradiation with an 808 nm laser (2 W/cm^2^, 80 s), MDA-MB-231/HER2 cells co-cultured with CuS@BSA-NB2 NPs showed a more significant photothermal effect than CuS@BSA treatment (Figure 6B). In addition, 50 μg/ml CuS@BSA NPs and CuS@BSA-NB2 NPs were used to treat MDA-MB-231/HER2 cells respectively for PTT, then AM and PI labeled living and dead cells. The CuS@BSA-NB2 NPs treatment group had a more significant death effect than the CuS@BSA NPs group at 808 nm (1 W/cm^2^, 160 s) (Figures 6C,E) and 808 nm (2 W/cm^2^, 80 s) (Figures 6D,F). Therefore, CuS@BSA-NB2 NPs had a stronger HER2 targeting effect, and were more enriched in the cells with high expression of HER2, which led to a better therapeutic effect under 808 nm.

## Discussion

CuS nanoparticles are a kind of material with plasmon resonance properties. In the near-infrared range, CuS NPs have a wide spectrum absorption and can convert light energy into heat energy by absorbing near-infrared light in a short time and have a strong photothermal conversion efficiency (Marin et al., 2018). Therefore, CuS NPs have received more attention from researchers in the treatment of tumors due to their unique light-to-heat conversion properties (Cheng et al., 2020; Yu et al., 2020). In the synthesis of CuS@BSA NPs, BSA was used to improve the water solubility, biocompatibility, and stability of CuS nanomaterials, which made it easier for the nanoparticles to enter the cells. Furthermore, many studies have shown that the encapsulation with BSA can also promote the function of nanoparticles (Zhang et al., 2018; Ma et al., 2021; Radwan et al., 2021).

With the diversification of treatment methods, it is also more popular to use CuS as the basic carrier to wrap or to modify other materials to synthesize more new therapeutic agents to treat tumors (Gao et al., 2018; Curcio et al., 2021). For example, Li et al. (Li et al., 2021) synthesized CuS@MnO/HA NPs, and they used CuS to co-load doxorubicin (DOX) and indocyanine green (ICG) to induce photothermal effects, and HA modification to endow the system with CD44 receptor-mediated tumor-targeting properties. Finally, the delivery of chemotherapeutic drugs and photosensitizers promotes multimodal anti-tumor therapy. Therefore, synthesizing different materials in different ways can endow the materials with multi-functional properties, and they will show more excellent properties when applied to the treatment of tumors.

At present, breast cancer is a malignant tumor, and antibody-drug conjugates are currently one of the important therapy plans. There are some researches that showed that HER2 nanobodies had been applied to breast cancer therapy (Keyaerts et al., 2016; Deken et al., 2020; Xenaki et al., 2021). BSA was used to be an anchor to connect CuS@BSA and NB2 with a covalent bond to synthesize CuS@BSA-NB2 NPs. Similarly, an analogical method also was used in other studies (Gao, Sun, et al., 2018; Zhang, Guo, et al., 2018; Feng et al., 2021). Therefore, in the functional characteristics of CuS@BSA-NB2 nanoparticles, NB2 was used to establish the function of targeting and recognizing tumor cells with high expression of HER2, and CuS@BSA nanoparticles were used to exert photothermal effect to treat tumor cells to achieve the combined treatment program of photothermal therapy and targeted treatment.

MDA-MB-231 cell is a kind of human breast cancer cell with low HER2 expression. We constructed a stable cell line with overexpression of HER2 in MDA-MB-231 cells to make sure the cells with high and low expressions of HER2 are under the same genetic background (Prelich, 2012). However, some other studies also used natural high and low expressions of HER2 cells for their research (Martínez-Jothar et al., 2019; Deken, Kijanka, et al., 2020), which may lead the drugs to produce different effects on different cells. Moreover, we applied CuS@BSA-NB2 at concentration of 50 μg/ml to investigate the PTT efficiency on the breast cancer cells. The result also showed that CuS@BSA-NB2 NPs had a higher ingested amount and photothermal effect under less radiation time than CuS@BSA NPs. Therefore, we speculated that CuS@BSA-NB2 NPs were more advantageous for photothermal therapy targeting HER2-positive breast cancer cells.

## Conclusion

In summary, the prepared CuS@BSA-NB2 NPs have shown excellent photothermal treatment and targeting effects in MDA-MB-231/HER2 cells. Here, we found that CuS@BSA-NB2 NPs had a perfect photothermal conversion efficiency and photothermal stability, including low toxicity, high targeting effect, and better photothermal treatment effects in MDA-MB-231/HER2 cells. Under the irradiation of an 808 nm laser, the temperature increase of CuS@BSA-NB2 nanoparticles was both concentration-dependent and time-dependent. Furthermore, the low concentration of CuS@BSA-NB2 NPs incubated within 24 h had almost no toxicity up to 100 μg/ml in MDA-MB-231/HER2 cells, indicating that the nanoparticles were biologically safe at a low concentration range. Besides, the photothermal effect of CuS@BSA-NB2 NPs was significantly stronger than CuS@BSA NPs at the same concentration in MDA-MB-231/HER2 cells, owing to its targeted recognition. Taken together, these results had shown the CuS@BSA-NB2 NPs have great potential to be as therapeutic agents for photothermal therapy by targeting HER2 in the future.

## Data Availability

The original contributions presented in the study are included in the article/Supplementary Material, further inquiries can be directed to the corresponding author.
