# Supplementary material for: CuS@BSA-NB2 Nanoparticles for HER2-Targeted Photothermal Therapy
Source: Front Pharmacol. 2022 Jan 21;12:779591. doi: 10.3389/fphar.2021.779591 (PMC8815789; doi:10.3389/fphar.2021.779591)
Supplement: Supplementary file 1 [file DataSheet3.docx]

Supplementary Material

## Supplementary Table

**Supplementary Table 1. The primers of HER2 and HER2 ECD for PCR**

| **Gene Name** | **Forward Primer (5'→3')** | **Reverse Primer (5'→3')** |
| --- | --- | --- |
| HER2 | ataagaatgcggccgccatggagctggcggc | ccgctcgagcactggcacgtccagaccca |
| HER2 ECD | acgcgtcgacaaatgacccaagtgtgcaccgg | ccgctcgagcgtcagagggctggctc |

## Supplementary Figures


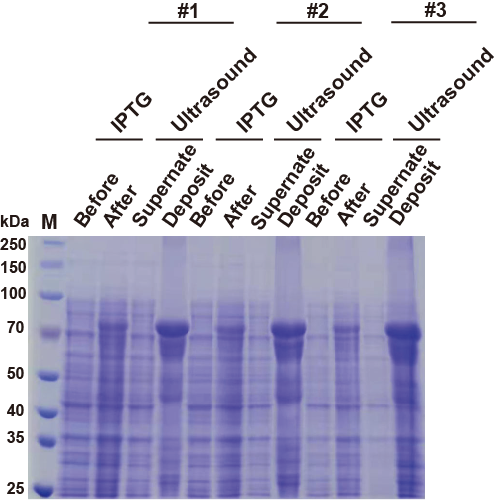


**Supplementary Figure 1.** SDS-PAGE image of the induction and expressions of HER2 (EDC). Three clones were picked to detect. The molecular weight of HER2 (EDC) was 72.9 kDa.


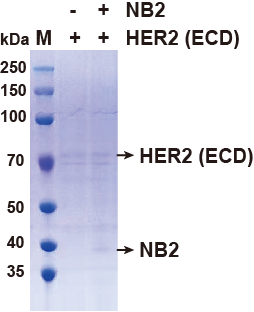


**Supplementary Figure 2.** SDS-PAGE image of co-incubation NB2 and supernatant of being induced and broken of HER2 ECD protein.


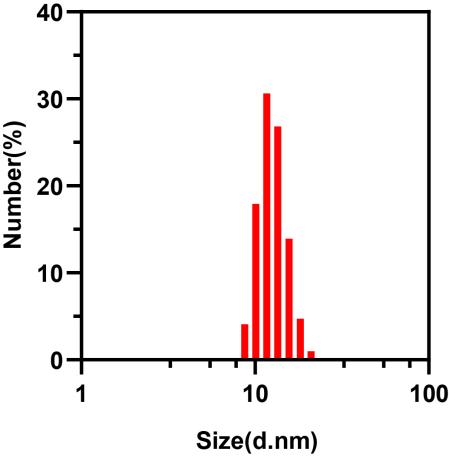


**Supplementary Figure 3.** DLS analysis of CuS@BSA NPs.


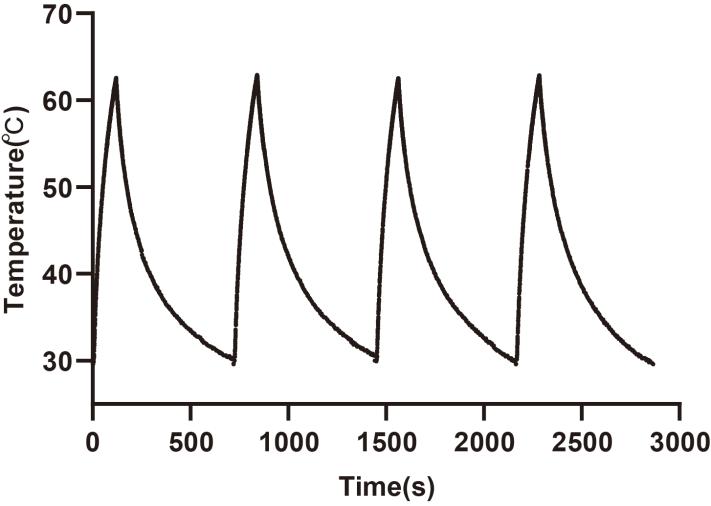


**Supplementary Figure 4.** The temperature curve of 200 μg/mL CuS@BSA NPs rised under 808 nm (1W/cm^2^) and cooled under room temperature for 4 cycles.
