# Supplementary figures and images for: CuS@BSA-NB2 Nanoparticles for HER2-Targeted Photothermal Therapy
Source: Front Pharmacol. 2022 Jan 21;12:779591. doi: 10.3389/fphar.2021.779591 (PMC8815789; doi:10.3389/fphar.2021.779591)

**Fig.2 C**

**
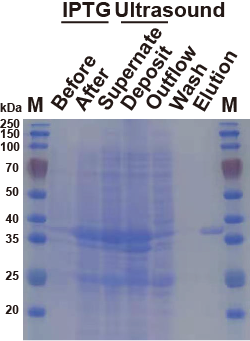
**
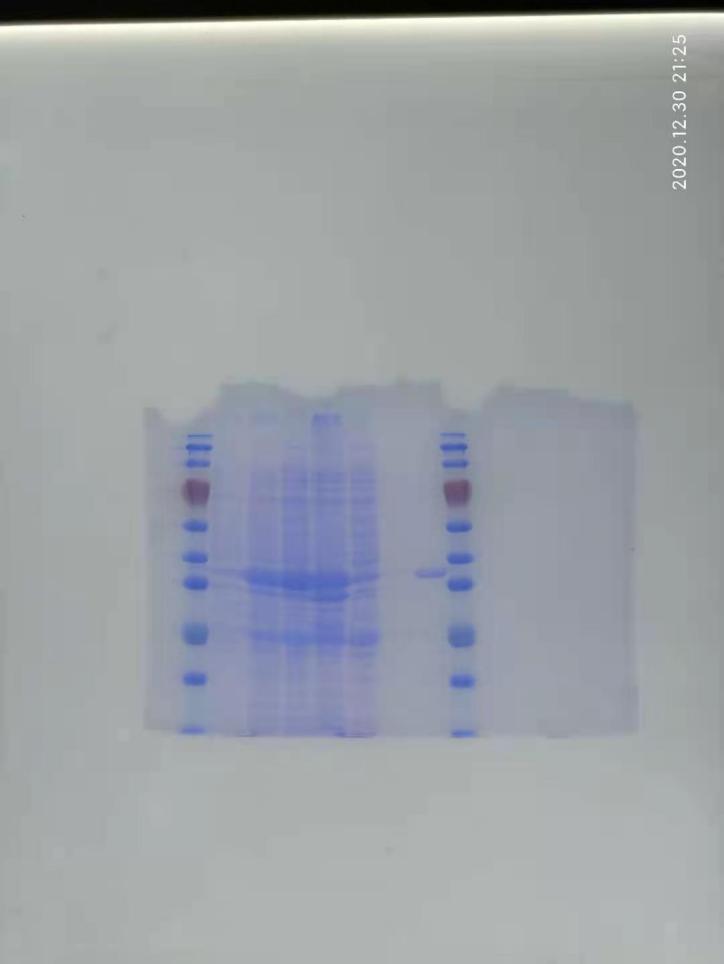


**Fig.2 D**

**
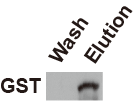

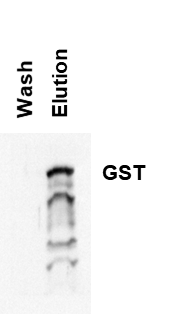
**

**Fig.2 E**

**
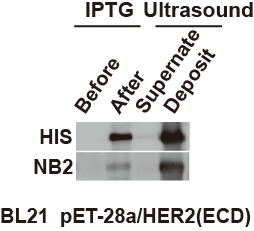

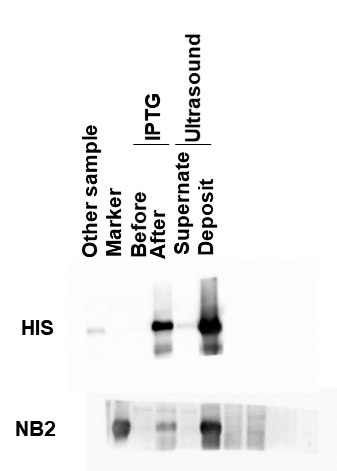
**

**Fig.2 F**

**
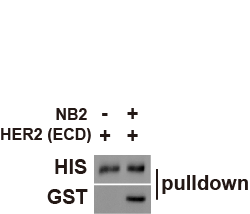

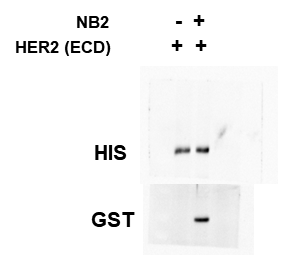
**

**Fig.3 B**

**
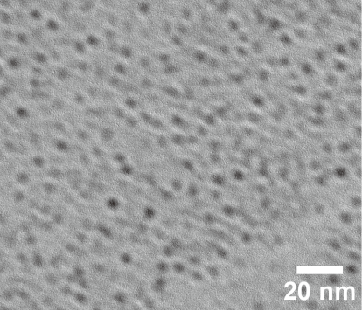

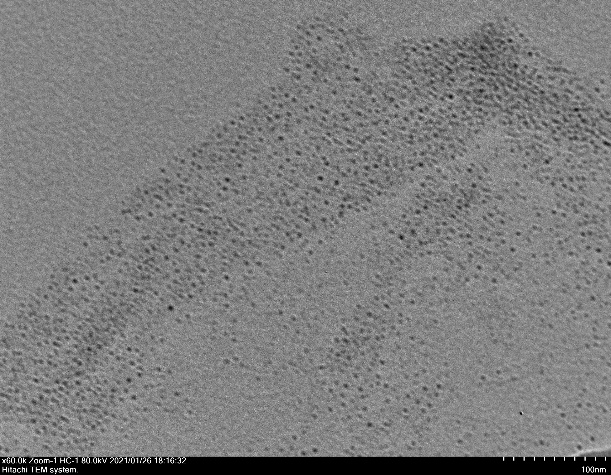
**

**Fig.3 C**

**
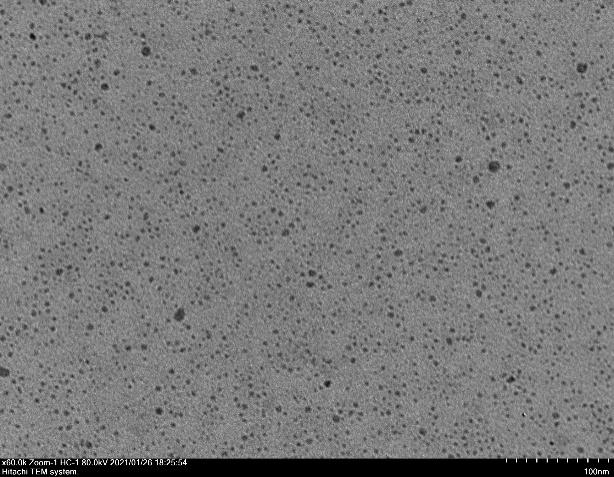
**

**Fig.4 A**


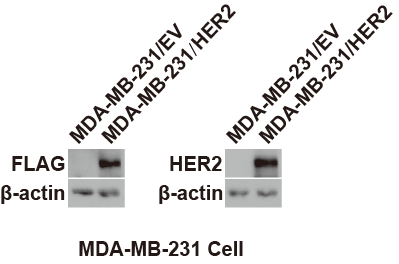

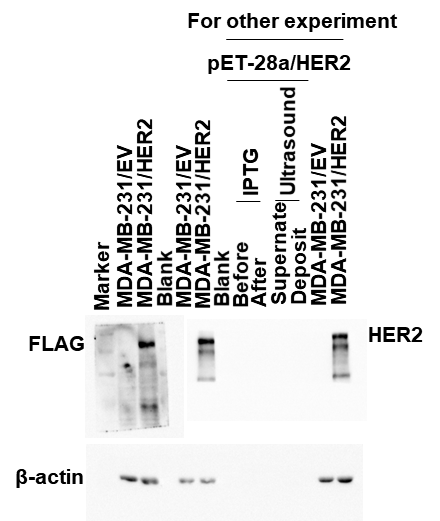


**Supplementary Figure 1**


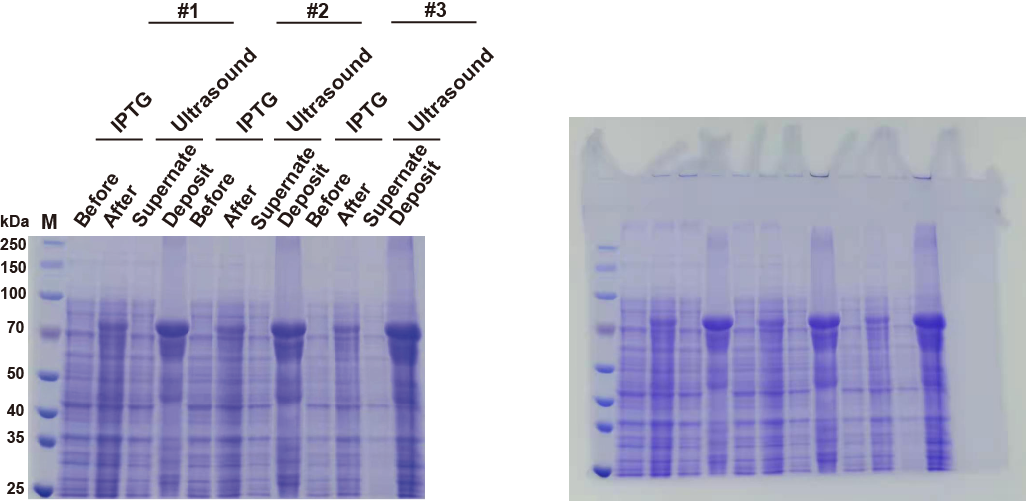


**Supplementary Figure 2**


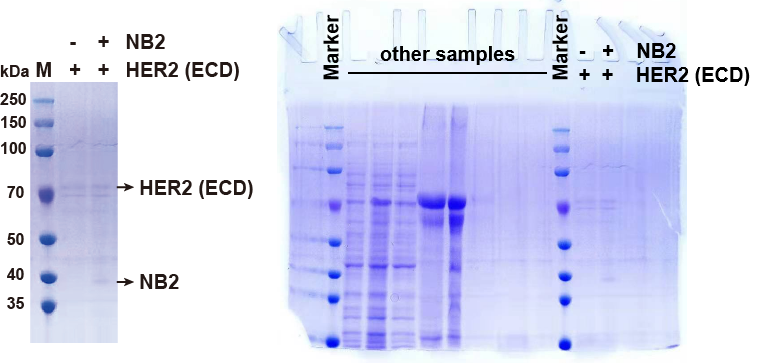

Supplement: Supplementary file 2 [file DataSheet1.docx]
